# Hospital Meetings, &c.

**Published:** 1899-11-25

**Authors:** 


### HOSPITAL MEETINGS, &C.

### CHELSEA HOSPITAL FOR WOMEN.

On Tuesday afternoon Lord Glenesk held a reception at the Chelsea Hospital for Women, of which he is the chairman. The occasion was the inspection of the improvements which have been planned and carried out with conspicuous success. The hospital has been repainted, fitted with electric light, electric lift, and hot-water service. The operating theatre has been remodelled and supplied with the most recent scientific appliances and conveniences, whilst an observation gallery has been admirably arranged for the convenience of the post-graduate students. The walls are of scaggliola, a substance resembling marble in appearance, and presenting a hard, polished surface. By means of the electric ventilating fan the whole air can be completely changed in a few minutes. In the sterilising room there is an additional apparatus for rendering the towels and operating garments of the surgeons aseptic. The nurses' home—the first improvement made in point of time—is much valued, and already paid for. Lord Glenesk welcomed his guests in a short speech, and gave an excellent account of the hospital finances. He said that the greater the need the higher the wave of philanthropy that rose to meet it, instancing the Prince of Wales's Hospital Fund as an example. The fears entertained on its promotion that the Fund would deplete the coffers of the individual hospitals had not been verified. He knew from personal experience of two hospitals which had reached a high water mark in their receipts since its inauguration. He trusted, therefore, that the vast contributions so rightly given to our soldiers and their families would not withdraw subscriptions from our home charities. Amongst the guests were the Marchioness of Headfort, Lady Vavasour, Lady Fox Young, Sir W. J. Colville, Sir Squire and Lady Bancroft, the Chief Rabbi (Dr. Adler), Major-General Wemyss, and many others.

